# Remote Cardiac Rhythm Monitoring in the Era of Smart Wearables: Present Assets and Future Perspectives

**DOI:** 10.3389/fcvm.2022.853614

**Published:** 2022-03-01

**Authors:** Anastasia Xintarakou, Vasileios Sousonis, Dimitrios Asvestas, Panos E. Vardas, Stylianos Tzeis

**Affiliations:** ^1^Department of Cardiology, Hygeia Group, Mitera Hospital, Athens, Greece; ^2^Heart Sector, Hygeia Hospitals Group, HHG, Athens, Greece; ^3^European Heart Agency, European Society of Cardiology, Brussels, Belgium

**Keywords:** smart wearable devices, remote monitoring, sensors, arrhythmia detection, heart rate, cardiac function

## Abstract

Remote monitoring and control of heart function are of primary importance for patient evaluation and management, especially in the modern era of precision medicine and personalized approach. Breaking technological developments have brought to the frontline a variety of smart wearable devices, such as smartwatches, chest patches/straps, or sensors integrated into clothing and footwear, which allow continuous and real-time recording of heart rate, facilitating the detection of cardiac arrhythmias. However, there is great diversity and significant differences in the type and quality of the information they provide, thus impairing their integration into daily clinical practice and the relevant familiarization of practicing physicians. This review will summarize the different types and dominant functions of cardiac smart wearables available in the market. Furthermore, we report the devices certified by official American and/or European authorities and the respective sources of evidence. Finally, we comment pertinent limitations and caveats as well as the potential answers that flow from the latest technological achievements and future perspectives.

## Introduction

Heart rhythm disorders are dominant public health issues, affecting more than 2% of the adult population. Their incidence is comparable to that of other major cardiovascular diseases, such as stroke, acute myocardial infarction and non-ischemic cardiomyopathy (1, 2). Cardiac rhythm abnormalities significantly increase with advancing age (1), so that the gradual aging of the world population has led to a sharp rise in the prevalence of cardiac arrhythmias, a phenomenon that is expected to intensify in the upcoming decades (3).

Atrial fibrillation (AF) is the most common cardiac arrhythmia, affecting approximately 46.3 million people worldwide. According to recent studies, in 2017, 10 million Europeans were suffering from AF, while, in the United States the number of the patients is expected to rise from 6 to 16 million, by 2050 (4, 5). The lifetime risk of AF is estimated at about 35% for Caucasians and 20% for African Americans (6). Even though sometimes asymptomatic (7), the disease causes significant morbidity, as it increases up to five times the risk of stroke (8), accounting for approximately one-third of all ischemic strokes (9, 10). Nevertheless, early arrhythmia diagnosis and initiation of anticoagulation treatment can lead to 64% stroke reduction (11).

Other rhythm disturbances, such as conduction disorders, bradyarrhythmias, supraventricular and ventricular tachycardias, induce significant morbidity and mortality, with related socioeconomic impact. These types of arrhythmias often result in patient hospitalization, while affected patients may experience severe symptoms, such as fatigue or syncope, as well as life-threatening events, or even sudden cardiac death (1).

Based on the above, early detection of cardiac arrhythmias is of paramount importance, in order to improve patient management. Timely diagnosis allows the implementation of appropriate interventions, either pharmacological or interventional, in order to prevent adverse effects, reducing morbidity and mortality. In the recently issued guidelines for the management of AF, the European Society of Cardiology recommends opportunistic screening in individuals aged ≥65 years to detect asymptomatic AF (12). Traditional methods of arrhythmia screening, such as electrocardiography (ECG) and continuous ambulatory Holter monitoring are mainly hampered by the limited period of rhythm recordings. Consequently, these tools are not useful for the screening of asymptomatic patients and the detection of paroxysmal arrhythmias, such as AF. Implantable loop recorders have drawbacks as well, since their cost may hinder their implementation in certain healthcare systems. Moreover, adverse events, such as skin erosion, infections, device oversensing or undersensing can limit their effectiveness (13).

Recently, rapid technological advances have led to the development of wearables with built-in micro-detectors, that can provide real-time monitoring of the vital signs and heart rate. Such devices can detect cardiac arrhythmias (14, 15), with varying accuracy, that depends on device type and detection method (16, 17).

The purpose of this review is to provide thorough insights into “smart” wearables, capable of cardiac rhythm monitoring, presenting the latest data derived from major clinical trials. The devices certified by the United States Food and Drug Administration (FDA), or CE-marked by the European Union authorities, are summarized, contributing to a comprehensive perception of the existing knowledge. Finally, legitimate concerns, based on literature evidence and future perspectives, are discussed, highlighting the limitations that need to be addressed and the aspects of potential development during the following years.

## Methods

The comprehensive review of the literature was achieved through screening of the Pubmed, Google Scholar and ClinicalTrials.gov databases from 1989 to January 2022, focusing mainly on articles published over the last decade. The searching procedure was based on several key terms regarding devices (“smart wearable devices” OR “smartwatches” OR “patches” OR “wristbands”) and heart conditions (“arrhythmia monitoring” OR “heart rhythm disorders” OR “cardiac diseases”), combined with Medical Subject Headings (MeSH). The first evaluation of the literature was based on the title and the abstract of each paper, while all the articles written in language other than English, or having included animal subjects, were dismissed. The reference section of the detected review articles was also probed and assessed, contributing to the overall selection of the literature. Finally, the Healthskouts Solutions Library for certified apps was used, as an additional source of certified smart wearable devices.

## Wearable Devices to Monitor Heart Rate and Cardiac Rhythm

Technological advancements have allowed heart rate sensors to be incorporated into numerous commercially available wearables. The spectrum of these devices ranges from smart accessories to sensors embedded into clothing and shoes. Patches, in particular, are leadless, wearable devices, that are attached to the patient's chest and provide ambulatory ECG monitoring over several days to weeks (18–21). Fitness bands and smartwatches are wrist-worn devices, able to track heart rate in real time (22–26). Heart rate sensors have also been integrated into accessories, such as rings (27, 28), necklaces (29), earbud headphones (30–32), chest straps (31, 33–35), footwear (36), glasses (37), even into textiles (38). Table 1 summarizes the most commonly used, FDA certified or/and CE-marked wearables to monitor heart rate, while Table 2 presents the essential technical specifications of each device.

**Table 1:** Wearable devices for heart rate and rhythm monitoring, certified by FDA or CE marked by the European authorities.

**Device type**	**Manufacturer**	**Product name**	**Cardiac function measurements**	**Other measurements**	**Certification**	**Official website**
Watch	Apple	Apple Watch series 7	HR, ECG	SpO2, physical activity, sleep tracker	FDA Certified, CE-marked	https://www.apple.com
Watch	Empatica	EmbracePlus	HR, HR variability	SpO2, skin temperature, respiratory rate, seizures detection	FDA Certified, CE-marked	https://www.empatica.com
Watch	Fitbit	Sense, Versa 2, Versa 3	HR, ECG	physical activity, sleep tracker, skin temperature, SpO2	FDA Certified, CE-marked	https://www.fitbit.com/global/us/home
Watch	Omron	HeartGuide	HR, BP	physical activity, sleep tracker	FDA Certified	https://omronhealthcare.com
Watch	Samsung	Galaxy Watch 4, Galaxy Watch Active2	HR, ECG	physical activity, VO2 max, fall detection	FDA Certified, CE-marked	https://www.samsung.com/global/galaxy/
Watch	Verily Life Sciences	Verily Study Watch	HR, ECG	electrodermal activity, inertial movements	FDA Certified	https://verily.com/solutions/study-watch/
Watch	Withings	Scanwatch	HR, ECG	SpO2, physical activity, sleep tracker	FDA Certified, CE-marked	https://www.withings.com/us/en/
Wristband	Biobeat	BB-613WP Wrist Monitor	HR, HR variability, BP stroke volume, cardiac output, cardiac index	SpO2, physical activity, respiratory rate, systemic vascular resistance, skin temperature	FDA Certified, CE-marked	https://www.bio-beat.com
Wristband	Empatica	Empatic E4	HR, HR variability	SpO2, skin temperature, respiratory rate, seizures detection	FDA Certified, CE-marked	https://www.empatica.com
Wristband	Fitbit	Charge 5, Luxe, Ace 3, Inspire 2	HR, ECG	physical activity, sleep tracker, skin temperature, SpO2	FDA Certified, CE-marked	https://www.fitbit.com/global/us/home
Chest monitor	Biobeat	BB-613WP Chest Monitor	HR, ECG, HR variability, BP, stroke volume, cardiac output, cardiac index	SpO2, physical activity, respiratory rate, systemic vascular resistance, skin temperature	FDA Certified, CE-marked	https://www.bio-beat.com
Patch	Bardy Diagnostics	BardyDx CAM	HR, ECG	None	FDA Certified, CE-marked	https://www.bardydx.com
Patch	BioTelemetry	ePatch	HR, ECG	None	FDA Certified, CE-marked	https://www.gobio.com
Patch	BioTelemetry	MCOT	HR, ECG	None	FDA Certified	https://www.gobio.com
Patch	Icentia	CardioSTAT	HR, ECG	None	CE-marked	https://www.icentia.com
Patch	InfoBionic	MoMe Kardia	HR, ECG	None	FDA Certified, CE-marked	https://infobionic.com
Patch	iRhythm	Zio Patch	HR, ECG	None	FDA Certified, CE-marked	https://www.irhythmtech.com
Patch	LifeSignals	WiPatch (1A Biosensor, 1AXe Biosensor, 1AX Biosensor)	HR, ECG	respiratory rate	FDA Certified, CE-marked	https://lifesignals.com
Patch	MediBioSense	Vital Patch, MBS HealthStream, MCM (Mobile Cardiac Monitoring)	HR, HR variability, ECG,	physical activity, respiratory rate, body temperature, fall detection, body posture	FDA Certified, CE-marked	https://www.medibiosense.com
Patch	Peerbridge Health	Peerbridge Cor	HR, ECG	None	FDA Certified	https://peerbridgehealth.com/for-physicians/
Patch	Preventice Solutions	BodyGuardian MINI	HR, ECG	None	FDA Certified, CE-marked	https://www.preventicesolutions.com/patients/body-guardian-heart
Patch	Rooti Medical	RootiRX	HR, ECG	skin temperature	FDA Certified	https://www.rootilabs.com
Patch	Samsung SDS	S-Patch	HR, ECG	None	CE-marked	https://www.samsungsds.com/en/cardio/cardio.html
Patch	Vpatch Cardio	Vpatch	HR, ECG	None	FDA Certified, CE-marked	https://www.vpatchcardio.com
Chest strap	NimbleHeart	Physiotrace Smart	HR, ECG	None	FDA Certified	https://www.nimbleheart.com
Chest strap	Qardio	QardioCore	HR, HR variability, ECG	physical activity, respiratory rate, skin temperature	FDA Certified, CE-marked	https://www.qardio.com
Chest strap/clothing	Equivital	eqO2+lifemonitor	HR, ECG, R-R interval	respiratory rate, skin temperature, galvanic skin response	FDA Certified, CE-marked	https://www.equivital.com
Chest strap/clothing	Medronic	Zephyr BioHarness	HR, ECG	physical activity, respiratory rate, skin temperature, body posture	FDA Certified	https://www.zephyranywhere.com/system/components
Chest strap/clothing	Nanowear	SimpleSense	HR	physical activity, respiratory rate, lung volume	FDA Certified	https://www.nanowearinc.com/simplesense
Chest strap/clothing	Nuubo	Nuubo System	HR, ECG	None	FDA Certified, CE-marked	https://www.nuubo.com/en-us
Clothing	HealthWatch Technologies	Master Caution	HR, ECG	respiratory rate, skin temperature, body posture	FDA Certified, CE-marked	https://healthwatchtech.com
Smart accessory	Õura	Oura Ring	HR	SpO2, skin temperature, sleep tracking	FDA Certified	https://ouraring.com
Smart accessory	toSense	CoVa 2	HR, HR variability, ECG, stroke volume, cardiac output	chest fluids, respiratory rate	FDA Certified	https://www.tosense.com

*BP, blood pressure; CE mark, Conformité Européene mark; ECG, electrocardiogram; FDA, Food and Drug Administration; HR, heart rate; SpO2, peripheral oxygen saturation; VO2 max, maximal oxygen consumption*.

**Table 2:** Main technical specifications of the smart wearable devices listed in Table 1. Presented information is derived either from the official website of the respective company, or from the user guide document, detected through the search engine UserManual.wiki.

**Devices**	**Sensor type**	**ECG channels**	Measurement range (accuracy)^*^	**Battery type**	**Recording time**	**Service life**
Apple Apple Watch series 7	Accelerometer, altimeter, ambient light sensor, blood oxygen sensor, electrical heart sensor, emergency SOS, gyroscope, optical heart sensor	1	HR: 30–210 bpm (accuracy not provided)	Rechargeable lithium-ion battery	18 h	~ 3 years
Empatica EmbracePlus	Accelerometer, electrodermal activity sensor, gyroscope, skin temperature sensor (thermometer)	not recorded	Not provided	Rechargeable lithium-ion battery	48+ h	2 years
Fitbit Sense, Versa 2, Versa 3	Accelerometer, altimeter, ambient light sensor, blood oxygen sensor, electrical heart sensor, electrodermal activity sensor, gyroscope, optical heart sensor, skin temperature sensor (thermometer)	1	HR: 20–220 bpm (accuracy not provided)	Rechargeable lithium-ion polymer battery	6 days	1–3 years
Omron HeartGuide	Oscillometric pulse sensors for blood pressure measurement	Not recorded	SBP: 60–230 mmHg (± 3 mmHg), DBP: 40–160 mmHg (± 3 mmHg), HR: 40–180 bpm (± 5 %)	Rechargeable lithium-ion polymer battery	8 times/day	1–2 years
Samsung Galaxy Watch 4, Active2	Accelerometer, ambient light sensor, barometer, bioelectrical impedance analysis sensor, electrical heart sensor, geomagnetic sensor, gyroscope, optical heart sensor	1	Not provided	Rechargeable lithium-type battery	Not provided	Not provided
Verily life sciences verily study watch	Electrical heart sensor, electrodermal activity sensor, inertial movement sensor	1	Not provided	Rechargeable lithium-ion battery	7 days	Not provided
Withings Scanwatch	Accelerometer, multi-wavelength PPG heart rate/SpO2 sensor	1	HR: 30–210 bpm (accuracy not provided)	Rechargeable lithium-type battery	~30 days	Not provided
Biobeat BB-613WP Wrist Monitor	PPG sensor	not recorded	SBP: 60–250 mmHg (± 5 mmHg) DBP: 40–150 mmHg (± 5 mmHg) HR: 40–240 bpm (± 3 %)	Non-rechargeable lithium manganese dioxide	3 days	3 years
Empatica Empatic E4	Accelerometer, electrodermal activity sensor, PPG sensor, skin temperature sensor (infrared thermopile)	Not recorded	Not provided	Rechargeable lithium-ion battery	24–48 h	Not provided
Fitbit Charge 5, Luxe, Ace 3, Inspire 2	Accelerometer, ambient light sensor, blood oxygen sensor, electrical heart sensor, electrodermal activity sensor, optical heart rate monitor, skin temperature sensor (thermometer), vibration motor	1	BP: 30–220 bpm (accuracy not provided)	Rechargeable lithium-ion polymer battery	7 days	Not provided
Biobeat BB-613WP Chest Monitor	PPG sensor	1	SBP: 60–250 mmHg (± 5 mmHg) DBP: 40–150 mmHg (± 5 mmHg) HR: 40–240 bpm (± 3 %)	Rechargeable lithium-ion polymer battery	6 days	3 years
Bardy Diagnostics BardyDx CAM	ECG electrodes	1	No range limitation	Not rechargeable lithium primary (coin cell) battery	7 days	2 years
BioTelemetry ePatch	ECG electrodes	1, 2, or 3	No range limitation	Rechargeable lithium-ion battery	5 days	2 years
BioTelemetry MCOT	ECG electrodes	2	No range limitation	Rechargeable lithium-ion battery	Not provided	3 years
Icentia CardioSTAT	ECG electrodes	1	No range limitation	Not provided	14 days	18 months
InfoBionic MoMe Kardia	ECG electrodes	2	No range limitation	Rechargeable lithium-ion battery	24 h	Not provided
iRhythm Zio Patch	ECG electrodes	1	No range limitation	2 lithium manganese dioxide coin cells gateway battery 1 lithium polymer cell battery	14 days	One-time use
LifeSignals WiPatch (1A Biosensor, 1AXe Biosensor, 1AX Biosensor)	ECG electrodes 1AX Biosensor: accelerometer, skin temperature sensor, gyroscope	2	HR: 30–250 bpm (± 3 bpm)	zinc-air battery (1A Biosensor), lithium-manganese dioxide battery (1AXe Biosensor, 1AX Biosensor)	3 days (1A Biosensor), 7 days (1AXe Biosensor), 5 days (1AX Biosensor	One-time use
MediBioSense Vital Patch, MBS HealthStream, MCM (Mobile Cardiac Monitoring)	accelerometer, ECG electrodes, skin temperature sensor	1	HR: 30–200 bpm (< ± 5 bpm)	Zinc Air battery	7 days	Not provided
Peerbridge Health Peerbridge Cor	ECG electrodes	2	No range limitation	Not provided	7 days	Not provided
Preventice Solutions BodyGuardian MINI	accelerometer, ECG electrodes	1–3	No range limitation	Rechargeable lithium-ion battery	16 days	Not provided
Rooti Medical RootiRX	ECG electrodes skin temperature sensor	1	No range limitation	Rechargeable lithium-ion polymer battery	7 days	1 years
Samsung SDS S-Patch	ECG electrodes	1	No range limitation	Not rechargeable lithium primary (coin cell) battery	5 days	2 years
Vpatch Cardio Vpatch	ECG electrodes	3	No range limitation	Not rechargeable lithium primary (coin cell) battery and rechargeable lithium-ion battery	7 days	5 years
NimbleHeart Physiotrace Smart	ECG electrodes	1	No range limitation	Not provided	Not provided	Not provided
Qardio QardioCore	ECG electrodes	1	SBP + DBP: 40-250 mmHg (± 3 mmHg), HR accuracy: ± 5 %	Rechargeable lithium-ion battery	24 h	2 years
Equivital eqO2+lifemonitor	accelerometer ECG electrodes breathing rate sensor skin temperature sensor	2	HR: 25-240 bpm (accuracy not provided)	Not provided	48 h	Not provided
Medronic Zephyr strap/clothing	accelerometer ECG electrodes breathing rate sensor skin temperature sensor	1	HR: 25-240 bpm (± 1 bpm)	Rechargeable lithium-ion polymer battery	12–28 h	Not provided
Nuubo Nuubo System	accelerometer ECG sensor	2	Not provided	Rechargeable battery	30 days	Not provided
HealthWatch Technologies Master Caution	ECG sensor breathing rate sensor skin temperature sensor	3–12	Not provided	Rechargeable or disposable battery	12–48 h	Not provided
Õura Oura Ring	accelerometer PPG sensor negative temperature coefficient sensor for body temperature	Not recorded	Not provided	Rechargeable Lipo battery	4-7 days	Not provided
toSense CoVa 2	ECG sensor breathing rate sensor skin temperature sensor	1	Not provided	Not provided	Not provided	Not provided

* *As reported by the manufacturer*.

*bpm, beats per minute; DBP, diastolic blood pressure; ECG, electrocardiogram; HR, heart rate; mmHg, millimeters of mercury; PPG, photoplethysmography; SBP, systolic blood pressure; SpO2, peripheral oxygen saturation*.

**Figure 1:**
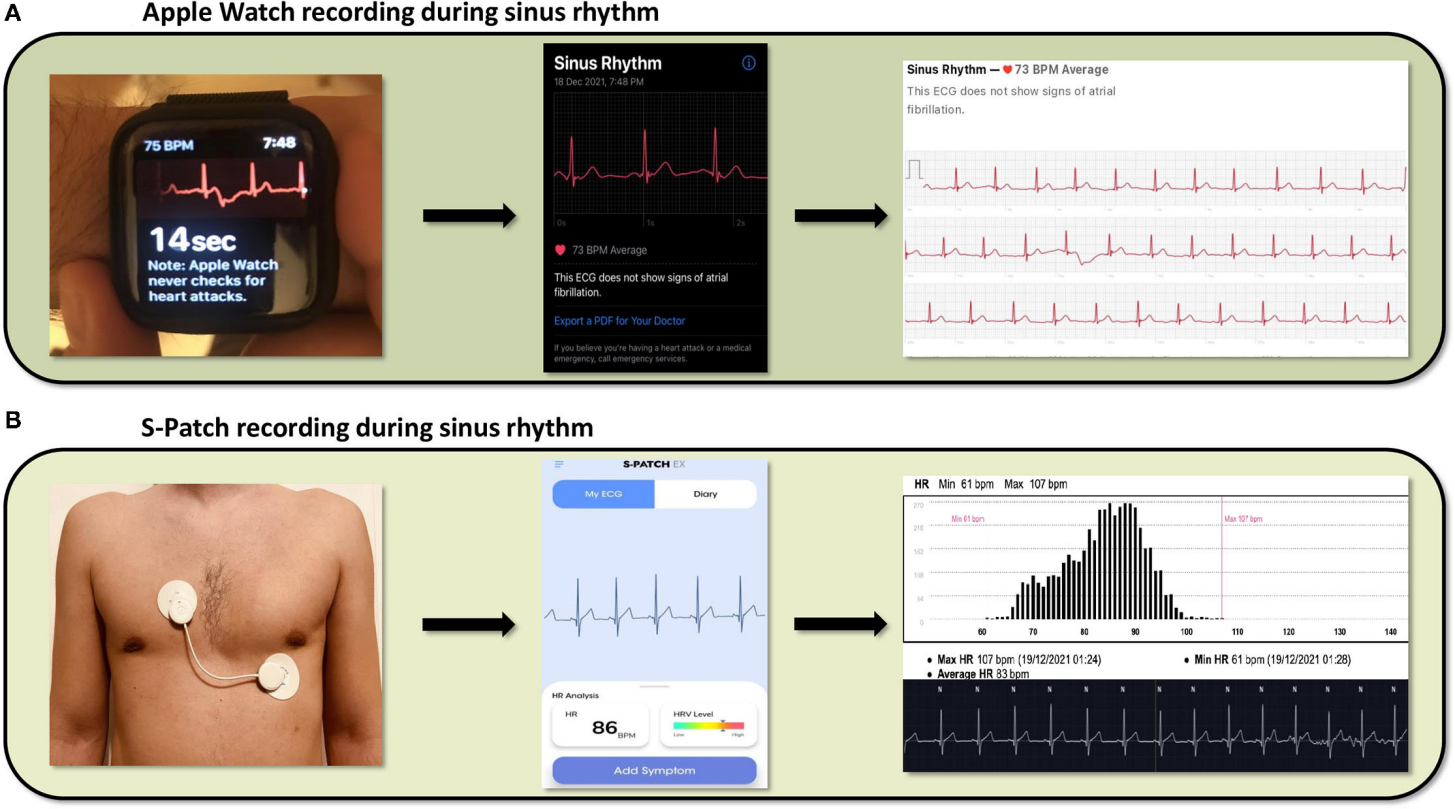
Cardiac rhythm recordings derived from a smartwatch (Apple Watch) and a patch (S-Patch), **(A)** From left to right, the images show the process of data collection from an Apple Watch, the ECG presentation in the respective smartphone application and the final report, featuring a possible diagnosis, **(B)** From left to right, the images show the same process captured by the wearable S-Patch.

Wearable devices rely on photoplethysmography (PPG) or single lead ECG tracings to detect heart rate. PPG is a non-invasive, optical technique that detects beat-to-beat alterations in the skin capillary bed volume (39). It utilizes a light source, usually a light-emitting diode, to shine light on the skin and a photodetector to measure the intensity of the non-absorbed light. Green light is most frequently used to minimize motion artifacts (40). Light attenuation correlates with the beat-to-beat volume changes of the microvasculature, caused by the peripheral pulse, thus allowing for heart rate assessment (39–41). Specific algorithms can identify heart rhythm irregularities, such as AF, based on fluctuations of the beat-to-beat interval (42–44). PPG technology is widely incorporated into the majority of the wearables used to monitor heart rhythm. On the other hand, patches use leadless electrodes and a sensor to obtain a single lead ECG, when attached to the patient's skin (45). Captured ECG tracings are reviewed to detect heart rhythm disturbances, either by automated algorithms, or by physicians (44). Of note, certain wearables, such as the Apple Watch series 4, or later, provide heart rhythm monitoring via both a PPG sensor and a single lead ECG (46). The latter can be recorded by wearing the Apple Watch and holding a finger of the opposite hand on the digital crown, creating an electric circuit that correspond to lead I of the 12-lead ECG. Figure 1 shows the recording of sinus rhythm by both a smartwatch and a chest patch.

The accuracy of the various wearables to monitor heart rate and detect cardiac arrhythmias is highly dependent on both the type of the device and the method of detection in use. In general, PPG-based heart rate measurements from wrist-worn devices show high agreement with those derived from simultaneous ECG tracings (34), although deviations have been reported in AF patients with high heart rates (47). Depending on the device model and the form of physical activity, the error in heart rate measurements by PPG ranges from 1.8 to 8.8% (24). Regarding AF detection, wearables using PPG signals are reported to have an accurary of 95–97% (48–50). In case of inconclusive readings, the diagnostic accuracy can be improved in devices providing ECG tracings, when the latter are interpreted by a trained physician (17, 44, 51). Patches show a consistently high accuracy in arrhythmia detection, comparable with that of ECG Holter monitors (52, 53).

## Wearables for Detection of Atrial Fibrillation

Table 3 summarizes the clinical trials and studies, which were conducted to evaluate the heart rhythm monitoring-oriented features of several smart wearable devices, cited in Section Wearables for Detection of Atrial Fibrillation.

**Table 3:** Comprehensive presentation of clinical trials and studies, conducted to evaluate the function and cardiac features of the wearable smart devices.

**References**	**Device type**	**Device name**	**Used technology (PPG vs ECG)**	**Number of recruited patients**	**Mean age (years)**	**Median monitoring time**	**Findings**
Rothman et al. (54)	patch	MCOT	ECG	305	56	25–30 days	The MCOT is superior to standard cardiac loop recorder regarding cardiac arrhythmia diagnosis
Tayal et al. (55)	patch	MCOT	ECG	56	66 ± 11	21 days	The MCOT showed high detection rate of AF in symptomatic patients after cryptogenic TIA/stroke
Miller et al. (56)	patch	MCOT	ECG	156	68.5	up to 30 days	The increased duration of monitoring with the MCOT is associated with a higher rate of paroxysmal AF detection
Rosenberg et al. (52)	patch	ZioPatch	ECG	74	64.5 ± 8.1	10.8 ± 2.8 days	Comparable estimation of the AF burden during the first 24 h between ZioPatch and Holter monitor. The longer recording duration achieved with ZioPatch resulted to increased diagnostic accuracy
Barrett et al. (57)	patch	ZioPatch	ECG	146	64	11 days	More arrhythmia events were detected with the ZioPatch compared to standard Holter monitor
Derkac et al. (58)	patch	MCOT, AT-LER	ECG	78,510	not provided	20 days: MCOT, 30 days: AT-LER	The MCOT showed higher diagnostic yield for arrhythmia detection compared to the AT-LER
Smith et al. (59)	patch	CAM	ECG	50	54.8 ± 17.8	24 h	Higher diagnostic accuracy and increased patient's comfortability are detected with the use of the CAM compared to standard 3-channel Holter monitor
Bumgarner et al. (44)	wrist-wearable	Apple Watch	ECG (Kardia Band technology)	100	68 ± 11	single tracing	The Kardia Band technology demonstrated 93% sensitivity and 84% specificity, regarding AF detection, compared to 12-lead ECG
Koshy et al. (60)	wrist-wearable	Fitbit smartwatch, Apple Watch	PPG	102	68 ± 15	30 min	Both smart devices showed a higher tendency to underestimated heart rate when AF was the leading cardiac rhythm
Rho et al. (61)	patch	ZioPatch (Zio-XT), CAM	ECG	29	73.1 ± 7.1	7 days	The CAM demonstrated more episodes of arrhythmia in combination with more accurate ECG recording. Patients' compliance was sufficient with both devices
Selvaraj et al. (62)	patch	VitalPatch	ECG	57	35 ± 11	not provided	The VitalPatch demonstrated a promising performance regarding physiological activity remote monitoring
Steinhubl et al. (63) (NCT02506244)	patch	iRhythmZio	ECG	2,659	72.4	up to 4 weeks	The intensive monitoring of high-risk patients, with a chest patch, contributes to increased rate of AF diagnosis
Tison et al. (64)	wrist-wearable	Apple Watch	PPG	9,750	42	20 min	The combination of smartwatch PPG technology and deep neural network, demonstrated 98% sensitivity and 90.2% specificity to identify AF, compared to standard 12-lead ECG
Ding et al. (65)	wrist-wearable	Samsung Simband 2	PPG	40	71	42 min	Data received from the wearable device, analyzed by a real-time algorithm, demonstrated high sensitivity (98.2%), specificity (98.1%) and accuracy (98. 1%) for irregular pulse detection
Guo et al. (66)	wrist-wearable	Honor Band, Huawei Watch	PPG	246,541	35	14 days	The PPG technology of the wearable devices could detect AF with a PPV of 91.6%
Kaura et al. (67)	patch	ZioPatch	ECG	116	70	14 days	Prolonged monitoring with the chest-patch was superior to the shorten Holter monitoring, regarding the detection of paroxysmal AF
Nault et al. (53)	patch	CardioSTAT	ECG	213	67 ± 11	24 h	The CardioSTAT showed high accuracy for AF diagnosis but moderate accuracy for atrial flutter diagnosis, compared to a Holter monitor
Pasadyn et al. (34)	wrist-wearable, chest strap	Apple Watch, Fitbit Iconic, Garmin Vivosmart HR, Tom Tom Spark 3, Polar H7	PPG	50	29	2 min	The Polar H7 chest strap demonstrated the highest accuracy to monitor heart rate among all wearables compared with the standard ECG
Perez et al. (68) (NCT03335800)	wrist-wearable, app	Apple Heart Study App and Apple Watch	PPG	419,927	41 ± 13	117 days	The individual tachogram demonstrated a PPV of 71% to detect AF, while the PPV of the irregular pulse notification was 84%
AI-Kaisey et al. (47)	wrist-wearable	Fitbit smartwatch, Apple Watch	PPG	32	68 ± 12	21 ± 1.3 h	Both devices demonstrated underestimation of the heart rate during AF
Inui et al. (69)	wrist-wearable	Apple Watch, Fitbit Charge	PPG	40	71	2 weeks	The work mode of the Apple Watch showed greater precision and accuracy to detect AF and measure heart rate, compared to the Fitbit wearable
Karunadas et al. (70)	patch	WebCardio	ECG	141	44.41	~24 h	Comparable accuracy of arrhythmia detection was observed between the WebCardio patch and the Holter monitor. However, 1^st^ degree AV block and PVCs could both be detected more accurately with the patch
Nachman et al. (71)	wrist-wearable	Biobeat BB-613WP	PPG	1,480	35.1 ± 23.8	single tracing	The device demonstrated agreement of 94.9% and 96.5% for hypertension and normal pressure, respectively, with the reference sphygmomano-meter-based device
Rajakariar et al. (51)	wrist-wearable	Apple Watch	ECG (Kardia Band technology)	218	67 ± 16	30 seconds	The Kardia Band technology demonstrated 94.4% sensitivity, 81.9% specificity and a PPV of 54.8% to detect AF. Improved diagnostic accuracy was observed with the combination of the device with an expert's interpretation
Schuurmans et al. (72)	wrist-wearable	Empatica E4	PPG	15	15	~5 min	Empatica E4 is comparable to the gold standard recording method for heart rate estimation
Avram et al. (73)	wrist-wearable chest patch	Samsung Galaxy Active 2, Biotel ePatch	PPG and ECG	204	62 ± 11.6	4 weeks	The collaborative function of the PPG and ECG sensors of the smart devices demonstrated high sensitivity (96.9%) and specificity (99.3%) for irregular heart rhythm monitoring
Caillol et al. (74)	wrist-wearable	Apple Watch	ECG	256	66 ± 6	single tracing	The Apple Watch was accurate to detect bradyarrhythmias and tachyarrhythmias, beyond AF and demonstrated high specificity but low sensitivity to detect ischemic heart disease
Ha et al. (75) (NCT02793895)	patch	SEEQ, CardioSTAT	ECG	336	67.4	30 days	Increased rate of postoperative AF detection in patients at high risk of stroke, by 17.9%, was observed using a 30 days continuous ambulatory cardiac rhythm monitoring system
Lubitz et al. (76)^*^ (NCT04380415)	wrist-wearable	Fitbit fitness tracker or smartwatch	PPG	455,699	47	not provided	An irregular heart rhythm detection by the Fitbit device had a PPV of 98.2% for AF diagnosis

*AF, atrial fibrillation; AT-LER, Autotrigger Looping Event Recorder; AV, atrioventricular; CAM, Carnation ambulatory monitoring; ECG, electrocardiogram; MCOT, Mobile cardiac outpatient telemetry; PPG, photoplethysmography; PPV, positive predictive value; PVCs, premature ventricular contractions*.

* *At the time of writing “The Fitbit Heart Study” had demonstrated its main outcomes only as a conference presented abstract*.

### Smartwatches/Wristbands

Smartwatches and wristbands are the most popular type of wearable devices, holding the dominant share of the global market, with a projected compound annual growth rate of 20% until 2026 (77). Inevitably, the wide spread of smartwatches that incorporate heart rhythm sensors among the population, raises queries about their role in AF screening and diagnosis (50), which is still controversial (78). At the same time, major studies are conducted, in order to define the accuracy of different smartwatch models to detect AF (50).

#### Apple Watch

The Apple Heart Study was one of the primary and most important studies regarding ambulatory ECG monitoring with the use of a wearable device (68). By recruiting 419,297 individuals without clinical history of AF, the authors examined the abnormalities of cardiac rhythm detected by the Apple Watch, in relation to AF detection, using an ECG patch, which was offered to those patients who received an irregular pulse notification from the device. Despite the limited number of the participants who finally returned the ECG patches and completed the study, the notification algorithm of the device had a positive predictive value of 84% to identify AF (95% confidence interval, 76% to 92%). In addition, a greater proportion of individuals older than 65 years was notified due to an irregular pulse, thus identifying a specific population group that could potentially benefit the most from AF screening with a smart wearable. Other studies with more restricted sample sizes have also assessed the accuracy of the Apple smartwatch to distinguish between AF and sinus rhythm, with comparable and promising results, regarding sensitivity and specificity of the embedded diagnostic algorithm (44, 64). However, physicians' involvement is necessary to provide an accurate diagnosis in unclassified recordings, which account for a significant proportion of the total tracings (51).

#### Fitbit Wearables

Recently, The Fitbit Heart Study (76) demonstrated a positive predictive value of 98% of the homonymous software algorithm to detect AF (79). The study enrolled more than 455,000 individuals, without history of AF, while an irregular heart rhythm was detected only in 4,728 (1%). The median population age was 47 years and people aged 65 years or older accounted for 12% of the total cohort. Among this elderly group of participants, the positive predictive value was also high (97%), encouraging the application of this technology to individuals older than 65 years of age, who usually have more comorbidities and are at greater risk of stroke.

Fitbit and Apple smartwatches have also been compared with the standard ECG, concerning the accuracy of the PPG technology to estimate heart rate. Two studies that included 102 and 32 participants, respectively, recorded a total of more than 91,000 heart rate values and showed that both devices underestimate heart rate during AF, especially when a rapid ventricular response of 100 beats per minute or faster occurs (47, 60). In addition, more accurate recordings were observed during night-time, when the physical activity is usually reduced, thus avoiding movement artifacts (47). In terms of direct comparison, the Apple Watch has a slightly superior diagnostic performance, compared to Fitbit smartwatches, closer to that of the gold standard ECG (34, 69).

#### Other Smartwatches/Wristbands

Many other companies have also launched smartwatches capable of heart rhythm detection, using either a PPG sensor or a combination of PPG and single-lead ECG. Two recent studies have shown high reliability of the AF detecting algorithms embedded into Samsung smart devices (65, 73). According to the findings, PPG sensor enhanced with a warning signal, prompting for an ECG recording, significantly increased the sensitivity for AF detection to 96.9% and the specificity to 99.3%. Thus, the high rate of specificity makes these wearables even more efficient for screening of the general population. Additionally, the algorithm was able to determine AF burden, a parameter associated with the risk of ischemic stroke and systemic cardioembolic events (73, 80). The Scanwatch by Withings, is a device able of recording 1-lead ECG tracings and is under evaluation in two ongoing clinical trials (NCT04493749, NCT04041466), regarding AF detection, compared with the standard 12-lead ECG. Finally, Huawei smart watches were used for the screening of patients included in the mobile Atrial Fibrillation II programme (mAFA-II programme), a two-phase trial, aiming to examine the optimization of AF screening and management through the integration of wearable PPG-based technologies (81). The early phase, called the Huawei Heart Study, assessed the effectiveness of smart wearables to detect AF, demonstrating a positive predictive value of 91.6% (66). The late phase, known as the mAFA II trial, investigated the value of a holistic care approach in AF patients, including the AF Better Care pathway (ABC pathway), combined with mobile smart technologies (82). However, these wearables have been certified only by the Chinese National Medical Products Administration.

Other wearables are able to track dominant factors of cardiovascular function, associated with arrhythmia initiation and development, even though they do not feature specific AF detection algorithms. The E4 wristband and EmbracePlus smartwatch by Empatica, in particular, assess the heart rate variability, a measure of the autonomic nervous systems related to AF development (83, 84). Schuurmans et al. validated the performance of Empatica E4 wristband in assessing heart rate variability, underlying the need for the user to remain still, in order to achieve accurate measurements (72). The HeartGuide smartwatch by Omron and the BB-613WP wristband by BioBeat Technologies feature blood pressure measurement, physical activity and sleep tracking, in addition to heart rate measurement (71, 85). Since the above are considered factors for AF development, these devices could potentially contribute to improved patient monitoring, individualized arrhythmia treatment and potentially reduction of AF burden (86).

### Patches

Wearable ECG patch monitors are appealing for AF detection, given their potential to store ECG tracings for a longer time, compared to conventional 24-h ECG Holter monitors. In general, patches provide an attractive alternative to conventional ambulatory Holter ECG monitoring for AF detection. They are easy to use and apply (63), less cumbersome than a Holter monitor (53, 57) and they interfere less with everyday activities, due to their leadless nature. Patients have reported to find patches comfortable and to prefer them over traditional Holter monitors (57), resulting in higher compliance. They can be worn for several days, rendering them ideal for mid-term rhythm monitoring, which increases the diagnostic yield of AF (63, 67). Physicians, on the other hand, believe that patch monitors provide definite diagnosis more often than a Holter monitor (57).

#### ZioPatch

The ZioPatch (iRhythm Technologies, USA) is a leadless, adhesive cardiac monitor, that is placed on the anterior chest wall by a technician, or easily self-applied by the patient, to provide up to 14 days of continuous ECG monitoring (87). After the completion of the monitoring period, the device is mailed back to the data processing center for the captured tracings to be processed, using an FDA cleared algorithm to detect potential arrhythmic episodes. Trained technicians review and classify the detected arrhythmias to generate a report that is then reviewed by the ordering physician (18). In a study by Rosenberg et al., the ZioPatch detected all AF episodes recorded in a 24-h ECG Holter monitor and reported similar AF burden rates, in patients simultaneously wearing both devices (52). In the mSToPS trial, 2,659 individuals at high risk of AF were randomly assigned to an immediate, 4-month, monitoring period that featured a total of 4 weeks of ZioPatch application, or to delayed monitoring, comprising of 4 months of usual of care, before starting a 4-month monitoring period, using the ZioPatch for a total of 4 weeks (88). At the end of first 4-months, the incidence of newly diagnosed AF was 4 times higher in the immediate monitoring group, compared to those allocated to usual of care for the corresponding time period. Over a 1-year follow-up period, 6.7 new AF cases per 100 person-years were detected in the total population of actively monitored participants, compared to 2.6 new AF diagnosis per 100 person-years in a matched observational control group. Active monitoring was also associated with increased likelihood of anticoagulant and antiarrhythmic therapy initiation, cardioversion procedures, ablation and increased health care resources utilization (63). ZioPatch was also found to be superior to short-term ECG Holter monitoring in detecting AF in patients after an ischemic stroke or transient ischemic attack (67). It should be noted that time to first AF detection with the use of the ZioPatch (and long-term rhythm monitoring in general) is inversely proportional to patient's AF burden; the higher the arrhythmic burden, the shorter the time to AF detection will be (18). The median duration to first AF detection reported in the mSToPS trial was 2 days (63).

#### Carnation Ambulatory Monitor

The Carnation Ambulatory Monitor (CAM, BardyDx, USA) is an adhesive patch monitor that is placed along the sternum for optimized P-wave capture. Improved P-wave clarity is associated with more accurate rhythm identification, compared to standard Holter monitoring (59). In a small study comparing the Carnation Ambulatory Monitor with the ZioPatch, in patients undergoing cardiac rhythm monitoring with the two devices for 7 days, patients with AF episodes during the monitoring period were successfully identified by both patches (61).

#### Mobile Continuous Outpatient Telemetry

The Mobile Continuous Outpatient Telemetry (MCOT) (BioTelemetry Inc, USA) consists of a sensor, an adhesive patch and a monitor and can be used for medium-term heart rhythm monitoring. The sensor is attached on the patch, which is then placed on the patient's chest. Each patch lasts for approximately 5 days, before it is replaced by a new one, until the desired monitoring period is complete. The sensor captures a two-lead ECG tracing, using the patch embedded electrodes. Data are transmitted via Bluetooth to the monitor, which constantly analyses the ECG, using algorithms based on pre-specified criteria. If a heart rhythm disorder is detected, or the patient marks a symptom, the monitor instantly transmits the ECG tracing to the central monitoring station and the referring physician is notified (89). Thus, the device offers near continuous, real-time, heart rhythm monitoring, that is only interrupted for sensor recharging. The MCOT monitor is able of detecting AF (89) with a higher diagnostic yield, compared to loop event recorders, especially for asymptomatic AF episodes (54, 58). In studies assessing extended rhythm monitoring in patients with cryptogenic stroke, AF was diagnosed in 17–23% of the participants using the MCOT system, with the rates of AF detection constantly increasing within the monitoring period (55, 56). The device has also been studied in patients undergoing radiofrequency ablation for AF, to detect arrhythmia recurrences during the follow-up period (90). A non-telemetry version of this monitor, the ePatch (BioTelemetry Inc, USA), is also commercially available. The device can be worn for up to 14 days and needs to be sent back to the manufacturer for data acquisition and review (91).

#### CardioSTAT

The CardioSTAT (Icentia Inc, Canada) is a single-lead device, worn on the upper chest to provide up to 14 days of heart rhythm monitoring through a lead I–like electrode configuration. In a validation study by Nault et al., the agreement between CardioSTAT and Holter monitor readings on AF detection was very high (53). In patients following cardiac surgery, a high-risk population for arrhythmic events, post-operative AF detection was increased by more than ten times, when individuals were continuously monitored with the CardioSTAT patch, compared to those assigned to usual care (75).

#### WiPatch

The WiPatch sensors (LifeSignals Inc, USA) are worn on the upper left part of the chest and record a two-lead ECG, for up to 72 h. Data are automatically transmitted to a connected mobile device and then uploaded to a cloud server for storage and analysis. Similar AF detection rates were reported in ambulatory patients monitored for 24 h, simultaneously by both a Holter ECG and a WiPatch (70).

#### VitalPatch

The VitalPatch (MediBioSense Ltd, UK) is another peel-and-stick device, capable of real-time heart rate monitoring. The device consists of a patch and a relay device (either a tablet or a phone). A single-lead ECG tracing is continuously recorded by a biosensor embedded in the patch, which is worn on the upper-left chest. Acquired data are transmitted to the relay device, analyzed and then sent to a central workstation. Apart from heart rate and single-lead ECG, VitalPatch also records data regarding heart rate variability, respiratory rate, body temperature and body motion, while it can also detect falls, providing a holistic telemonitoring of physiological measurements and body activity (62). Recently, the device was updated with arrhythmia detection, including AF, but clinical data are still lacking.

### Clothing and Accessories

The integration of heart rate sensors into textiles and accessories has led to the development of a wide variety of devices, that can track heart rate and/or record ECG. Accessories, such as rings (27, 28), headphones (30, 32), and footwear (36) are reported to be reliable in heart rate measurements, even though their accuracy can be compromised during high intensity exersice (31). Another monitoring system features a devices that resembles a necklace (CoVa monitoring system) (29), which, not only monitors heart rate and ECG in real-time, but also provides information regarding stroke volume, cardiac output and fluid status, providing an holistic hemodynamic assessement of heart failure patients, rather than merely cardiac rhythm monitoring. Chest straps, like the Zephyr BioHarness (33, 35, 92–94), the Polar H7 (30, 34, 95) and the EQ02 Lifemonitor (96, 97) provide a wide range of biomeasurments and are used mainly in athlete training, even though the Polar H7 chest strap has been utilized as an AF screening tool, with high accuracy (98). Sensors embedded in clothing, such as the Nuubo vest (99, 100) and the Master Caution shirt (101), allow for continuous ECG monitoring, fascilitating arrhythmia detection. In a study by Pagola et al., prolonged patient monitoring, using the Nuubo vest, after a cryptogenic stroke, led to the diagnosis of AF in 20% of the participants (102). In general, smart clothing and accessories reliably provide a wide variety of biomeasurements, including heart rate, but validation as diagnostic tools for arrhythmia detection is lacking for most of the products under this category.

## Arrhythmias Other Than AF

Although all the large-scale studies conducted on the use of smartwatches as diagnostic and monitoring tools focus on AF, data in the literature demonstrate their potential contribution to the detection of arrhythmias other than AF (50). The spectrum of the heart rhythm disorders that can be detected with wearable devices is outlined in Table 4.

**Table 4:** Heart rhythm disorders identified by wearable devices and the respective modality used to detect them.

**Heart rhythm disorders**	**Smart wearable modality**
**Tachycardias**	
Sinus tachycardia	ECG
Supraventricular tachycardia	ECG
Ventricular tachycardia/fibrillation	ECG
**Bradycardias**	
Sinus bradycardia	ECG
Pause	PPG, ECG
**Ectopy**	
Supraventricular premature complexes	ECG
Ventricular premature complexes	ECG
**Irregular rhythms**	
Atrial fibrillation	PPG, ECG
**Atrioventricular conduction disorders**	
First degree AV block	ECG
Second degree AV block	ECG
Complete AV block	ECG
**Other**	
QT interval assessment	ECG

*AV, atrioventricular; PPG, photoplethysmography; ECG, electrocardiogram*.

### Smartwatches/Wristbands

Bradyarrhythmias and tachyarrhythmias can be identified with the use of smartwatches (74). The Apple Watch has been used in the detection of ventricular arrhythmias in two patients suffering from arrhythmogenic right ventricular cardiomyopathy and episodes of non-sustained ventricular tachycardia (VT), respectively, enabling physicians to correlate the reported symptoms with the underlying cause, which would otherwise remain undiagnosed (103). Captured single-lead ECG tracings of ventricular tachycardia in a patient with structural heart disease, or pre-excited AF with rapid ventricular response in an otherwise healthy young individual, using an Apple Watch, have also been reported (104). In patients wearing the Apple Watch, episodes of supraventricular tachycardia (SVT), such as atrioventricular reentrant and atrioventricular nodal reentry tachycardia have been recognized, as well (105, 106). Furthermore, researchers suggest an alternative use of these wrist wearables, by placing the sensor either at both upper extremities or at the abdomen and the chest in order to receive a more comprehensive recording that best resembles the standard 12-lead ECG. As a result, differential diagnosis between AF and atrial flutter could be facilitated. Furthermore, abnormalities associated with sudden cardiac arrest in young adults with ventricular pre-excitation, Brugada ECG pattern, arrhythmogenic right ventricular cardiomyopathy, hypertrophic cardiomyopathy or long-QT syndrome can also be detected (107, 108).

### Patches

Apart from AF, patches that monitor heart rhythm can identify a series of other clinically significant arrhythmias, being a useful tool in the diagnostic work-up of patients with symptoms such as palpitations, syncope or presyncope. Supraventricular tachycardia was the most common rhythm disorder identified in a large cohort of individuals monitored with the ZioPatch (18, 52), as well as in patients with symptoms of arrhythmia discharged from the emergency department (109). Prolonged rhythm monitoring with wearables other than the ZioPatch is also associated with an increased rate of SVT diagnosis (55, 56, 70, 89). Patches providing clear identification of the P-wave facilitate further classification of the detected SVTs (61). Côté et al., have reported two cases of Wolff-Parkinson-White syndrome with intermittent pre-excitation, not present in 12-lead ECG, in children monitored with the CardioSTAT patch, who were complaining of palpitations (110).

Among participants actively monitored in the mSToPS trial, the detection of significant pauses and high degree atrioventricular block, along with runs of non-sustained VT, resulted in increased rates of pacemaker and implantable defibrillator implantations (63). In comparative studies, the MCOT patch was superior to a loop event recorder in detecting bradycardia, cardiac pauses, sustained or symptomatic SVT, asymptomatic high ventricular rates and runs of VT (54, 58). In patients undergoing transcatheter aortic valve replacement (TAVR), prolonged, patch-based, rhythm monitoring, prior to the procedure, can detect significant bradyarrhythmias in one-fifth of the patients, some of which may require a change of treatment (111). Following TAVR, Tian et al., identified patients with late high degree atrioventricular block, using the BodyGuardian patch (Preventice Solutions, Inc, USA) (112). The same patch was also found to reliably assess the QT interval, both in healthy individuals and long QT syndrome patients and could be used to remotely monitor patients in risk of QT interval prolongation and arrhythmias (113).

## Limitations

**Figure 2:**
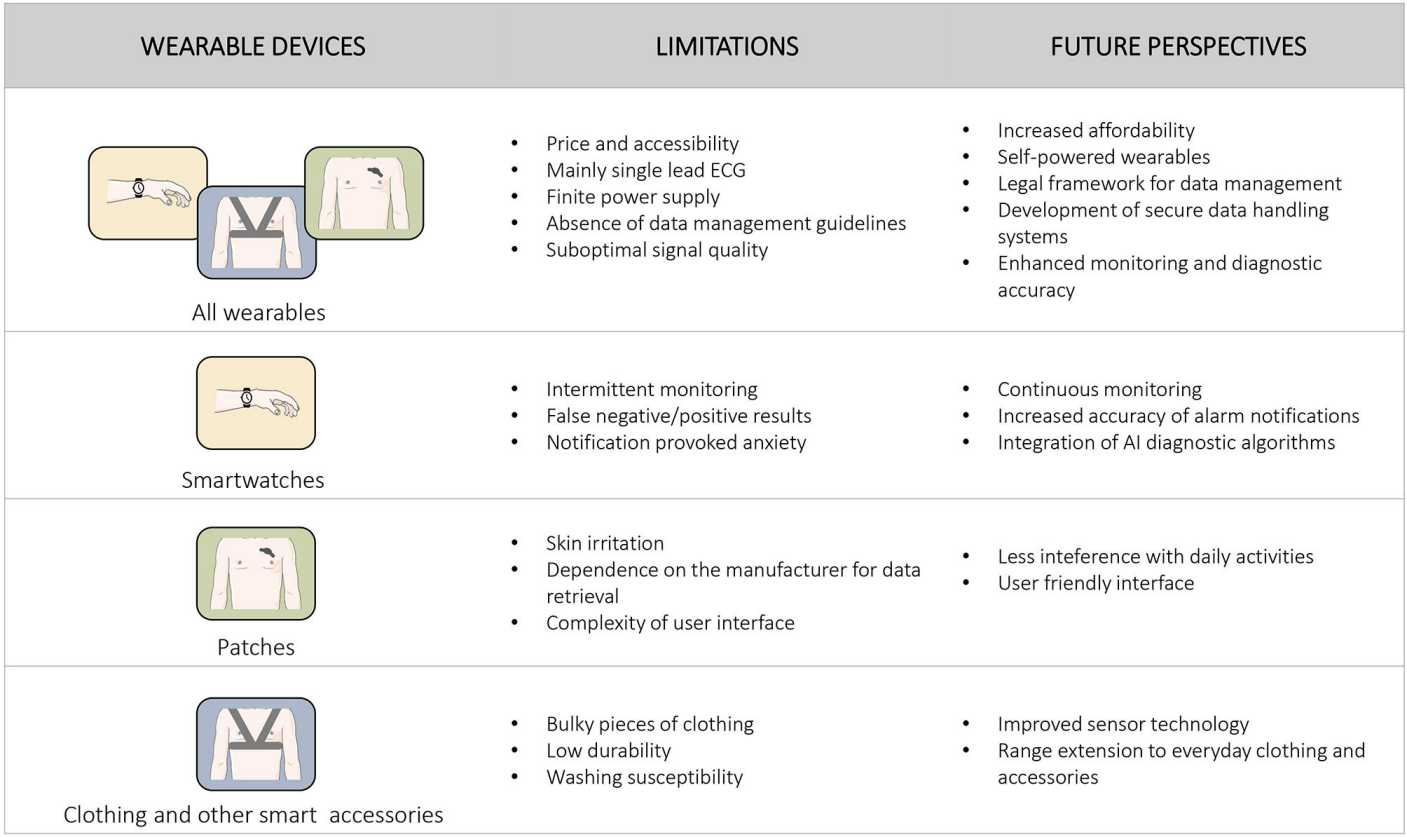
Main limitations and future perspectives of wearable devices for heart rhythm monitoring are presented. Specific information for each category of wearable devices is presented separately.

Despite the variety of benefits associated with wearable technology, several limitations have to be overcome in order to establish their role as medical devices for cardiac rhythm monitoring and diagnosis, in everyday clinical practice. Figure 2 summarizes the main limitations and future perspectives, concerning the growing population of smart wearables.

Smartwatches and wristbands are practical and able to track heart rhythm during every possible physical activity, using a semi-continuous PPG sensor, with or without intermittent ECG tracings, when available. Consequently, they lack the ability of continuous rhythm monitoring and require the cooperation of the user in order to achieve a reliable recording and capture the required data (12). Due to this operation mode, recording of paroxysmal arrhythmias may be missed, preventing accurate diagnosis and targeted management. In addition, the necessity for patient's alertness precludes monitoring during episodes with associated loss of consciousness, that could reveal malignant underlying arrhythmias, while the concomitant reduction of the peripheral blood pressure may weak the pulse signal, affecting the quality of the PPG signal (69). In view of this, in the WATCH AF trial, a high dropout rate of record files due to inadequate quality of the signal was observed, thus restricting the applicability of a smartwatch monitor in everyday practice (48). Smartwatches often demonstrate false positive results, occasionally leading to overdiagnosis and overtreatment of patients. At the same time, pathological signals and repeated notifications provoke intense anxiety to the user, often affecting both his physical and mental health (12).

On the other hand, wearable patches provide continuous rhythm recording during the application period, without requiring the active involvement of the user. Nonetheless, they manifest caveats that also restrict their applicability. Some patients may experience skin irritation, which is the most common adverse reaction of adhesive patch monitors (54, 63, 87). Noise recording could render the rhythm tracing uninterpretable, especially when single-lead patches are used (53). Another limitation is the fact that certain devices, such as the ZioPatch and the CardioSTAT, have to be mailed back to the manufacturer for data collection and analysis. This process could result in significant turnaround times between the end of the monitoring period and arrhythmia detection. Moreover, there is a clear dependance on the device company for data retrieval and analysis for most patch monitors, while the cost of using these devices is not negligible. Finally, user interface is quite complex for people who are not particularly familiar with handling novel technological platforms, excluding its utilization by elderly patients and non-familiarized physicians (114).

The integration of sensors into garments overcomes some of the drawbacks listed above, such as the need for the user to operate certain devices. On the other hand, incorporating electronics into fabric results in bulky, inflexible pieces of clothing. Moreover, the lifespan of smart clothing may be limited, affected by low durability and washing susceptibility.

Most of the wearables that capture ECG tracings provide data equivalent only to one of the 12 leads of the traditional ECG, thus limiting their value in detecting more complex arrhythmias and cardiac disorders (74). Furthermore, despite the significant progress observed in the field of lithium battery lifetime, power supply is still of finite duration, demanding repeated recharging and resulting to intervals of monitoring interruption (14). Besides that, the great abundance of sensitive data collected through these new technologies, require the development of strict policies to ensure safe storage, transparency, privacy and security of users' personal data (14, 115). Additionally, the crucial challenge of pricing and accessibility of smart wearables is still under discussion. Their consideration as medical devices establishes new standards that require the normalization of economic inequalities, in order to avoid health disparity. Moreover, the involvement of the public and private insurance systems in device reimbursement is unclear and is yet to be determined (14, 116).

Wearable devices have evidence-based credentials that establish their role as a screening tool for the detection of cardiac arrhythmias, especially AF, affecting decision making in patient pharmacological treatment. However, the benefit derived from the initiation of anticoagulation, based on AF diagnosis by wearable devices screening, has not been confirmed. In fact, despite the increased diagnostic rate of AF episodes, in high-risk individuals, in the STROKESTOP trial (117, 118) and the LOOP study (119), the LOOP study failed to show any added benefit, regarding the endpoint of stroke or systemic embolism, after initiation of preventive anticoagulation (119). These results imply that not all AF episodes in asymptomatic patients pose the same thromboembolic risk and underscore the need for further evaluation, in order to determine the exact role of AF screening and indicate specific AF characteristics and/or AF patient groups that derive potential benefit from oral anticoagulation initiation.

## Future Perspectives

Undoubtedly, novel technologies bear the potential to shift the paradigm of disease diagnosis and patient management. The ideal wearable device for heart rhythm monitoring should be easy to use, even by the elderly and less familiarized patients, not interfere with daily activities and provide continuous and accurate real-time heart rhythm monitoring. Most commercially available wearables are capable of continuous monitoring for several days, providing they have sufficient power. Use of self-powered technology would, theoretically, allow for uninterrupted, extended use of cardiac monitoring devices (120). Wearables with integrated mobile network access could instantaneously transmit data to central analysis stations, circumventing the need of a transmitting device, and potential data loss if the patient is not within the range of the latter, when a clinically significant arrhythmia appears (70). This would grant real-time surveillance of the heart rhythm, which is of outmost importance in high-risk patients, such as those in risk of ventricular arrhythmias.

Further improvement of the diagnostic accuracy has the potential to transform wearables, such as smartwatches, from screening and pre-diagnostic tools to diagnostic modalities. The integration of artificial intelligence algorithms in basic medical tools, such as the 12-lead ECG, has demonstrated promising results, regarding the early and accurate detection of structural heart disorders, thus extending beyond the field of arrhythmia diagnosis (121, 122). The combination of this technological advancement with wearable devices could improve the reliability of their measurements and provide prognostic features for the detection of subclinical cardiac conditions (123). Advances in deep learning technologies and their application in the diagnostic algorithms will certainly further increase their diagnostic accuracy (124, 125). Moving away from validation studies, clinical trials pursuing hard endpoints, such as cardiovascular mortality or stroke, are essential to facilitate wider acceptance of wearables from clinicians, their implementation in everyday clinical practice and to support device reimbursement from insurance companies.

As wearable devices become more affordable and reach an increasingly number of consumers, a plethora of sensitive health data are anticipated to be generated. There is a clear necessity for an integrated system to collect and safely store personal information, in a way that will ensure users' privacy. Clear legal regulations, along with an ethical framework, under which personal data are collected and handled, are essential (126). Data processing should target accurate diagnosis and provide the attending physician with clinically meaningful information. In this way, integrated data handling systems could translate to improved patient management, with personalized healthcare interventions and better utilization of health care resources.

## Conclusions

Wearable devices are a new reality in monitoring and management of cardiac arrhythmias. Pertinent caveats, such as signal quality, connectivity issues, battery life limitations, sub-optimal diagnostic accuracy and data security and storage, need to be addressed, in order to enable their full utilization as medical devices. However, advanced technological developments contribute to rapid improvement and accomplishment of future intentions and are expected to establish the role of smart wearables as important tools in the emerging era of telehealth, remote patients' control, personalized and precision medicine.

## Author Contributions

ST, AX, VS, DA, and PV: conception of the theme and design. AX and VS: data collection and preparation of the draft manuscript. ST: editing. ST and PV: final revision and supervision. All authors approved the final version of the manuscript.

## Conflict of Interest

The authors declare that the research was conducted in the absence of any commercial or financial relationships that could be construed as a potential conflict of interest.

## Publisher's Note

All claims expressed in this article are solely those of the authors and do not necessarily represent those of their affiliated organizations, or those of the publisher, the editors and the reviewers. Any product that may be evaluated in this article, or claim that may be made by its manufacturer, is not guaranteed or endorsed by the publisher.
